# Neoadjuvant chemotherapy followed by radical surgery reduces radiation therapy in patients with stage IB2 to IIA2 cervical cancer

**DOI:** 10.1186/s12957-022-02731-x

**Published:** 2022-08-23

**Authors:** Yuhui Huang, Lei Chen, Jing Cai, Lu Yang, Si Sun, Jing Zhao, Zhoufang Xiong, Zehua Wang

**Affiliations:** Department of Obstetrics and Gynecology, Union Hospital, Tongji Medical College, Huazhong University of Science and Technology, 1277 Jiefang Avenue, Wuhan, 430022 China

**Keywords:** Neoadjuvant chemotherapy, Locally advanced cervical cancer, Adjuvant radiotherapy, Prognosis

## Abstract

**Background:**

To investigate whether carboplatin-liposomal paclitaxel neoadjuvant chemotherapy (NACT) benefits patients with locally advanced cervical cancer (LACC) through avoiding or delaying postoperative radiation.

**Methods:**

A total of 414 patients with cervical cancer of International Federation of Gynecology and Obstetrics (FIGO 2009) stages IB2–IIA2 were included in the retrospective cohort study, who had received carboplatin-liposomal paclitaxel chemotherapy followed by radical surgery (NACT group) or primary radical surgery (PRS group) between 2007 and 2017 at our hospital. The baseline clinicopathological characteristics at diagnosis, postoperative pathological risk factors, and oncological outcomes after surgery, including postoperative radiation (as adjuvant treatment or treatment of recurrent diseases), progression-free survival (PFS), and overall survival (OS), were compared between the groups. Before treatment, the patients in the NACT group had significantly more advanced tumor stages and larger tumor sizes than those in the PRS group.

**Results:**

The NACT reduced the tumor volumes remarkedly with a response rate of 62.4%, and the tumors in the NACT group were smaller than those in the PRS group when the patients were subjected to radical surgery. Furthermore, postoperative pathology examination revealed less frequent deep stromal invasion in the NACT group than in the PRS group. According to the presence of pathological risk factors for recurrence, 54.82% of women in the NACT group needed adjuvant radiotherapy, while 60.87% in the PRS group, and in fact, 33.00% of NACT patients and 40.09% of PRS patients received adjuvant radiation. In addition, 8.12% of NACT patients and 9.68% of PRS patients underwent radiotherapy after relapse. The cumulative postoperative radiation rate was significantly lower in the NACT group (*P* = 0.041), while the differences in 5-year OS and PFS were not statistically significant between the groups.

**Conclusions:**

NACT reduces the pathological risk factors and the use of radiation without compromising survival in patients with LACC, which may protect younger patients from radiation-related side effects and subsequently improve the quality of life.

**Trial registration:**

ISRCTN Registry, ISRCTN24104022

## Introduction

Cervical cancer is a major cause of cancer mortality in women worldwide. According to global cancer statistics, there were an estimated 604,000 new cases and 342,000 cervical cancer-related deaths in 2020 [15]. While radical surgery is accepted as the standard treatment for early-stage cervical cancer, the optimal treatment for patients with stages IB2–IIA2 remains controversial.

Though concurrent chemoradiation is recommended as the standard treatment [12], it sometimes brings serious, non-reversible complications, such as bladder dysfunction, sexual dysfunction, and gastrointestinal dysfunction. On the other hand, most advanced cervical cancers occur in developing countries and areas, where the lack of radiotherapy equipment may limit the use of chemoradiation. Therefore, how to reduce the need for radiotherapy to improve the quality of patients’ life is still a challenge. Neoadjuvant chemotherapy (NACT) followed by radical surgery has been widely used for patients with locally advanced cervical cancer (LACC) to reduce tumor volume, improve the rate of resection, and control the potential micro-metastasis. Previous studies have also shown that NACT may have an impact on the clinicopathological risk factors and postoperative radiotherapy [11, 14, 18]. One phase III trial reported that NACT with BOMP (bleomycin, vincristine, mitomycin, and cisplatin) regimen before radical surgery significantly reduced both the proportion of patients who met the criteria for postoperative radiation and the proportion of patients who received postoperative radiation compared to primary radical surgery (PRS) [9]. Similar results were also reported in another multicenter study with NACT consisting of IP (irinotecan plus cisplatin) and TP (paclitaxel plus cisplatin) regimens [18]. However, Li et al. failed to identify these findings in their study with NACT of TC (paclitaxel plus carboplatin), TP, and PF (fluorouracil plus cisplatin) regimens [13], as well as another study with four different chemotherapy regimens [20]. In these studies, the NACT regimens were different, and the response rates varies from 58.8 to 75.7%; the inconsistent results impacted the credibility of the effect of NACT on postoperative radiotherapy. Therefore, the potential benefits of different regimens of NACT on postoperative adjuvant radiotherapy need to be tested further.

There have been no studies investigating the effect of NACT with carboplatin-paclitaxel on adjuvant radiation, which has been recognized as one of the most effective regimens with a high response rate of 95% and mild toxicity [3]. Thus, we performed this retrospective study to investigate whether NACT with liposomal paclitaxel and carboplatin reduces the pathological risk factors and postoperative radiation in patients with LACC and compare the oncologic outcomes between patients who underwent NACT followed by radical surgery and patients who underwent PRS.

## Methods

### Patients

All patients with International Federation of Gynecology and Obstetrics (FIGO 2009) stage IB2–IIA2 cervical cancer who underwent carboplatin-liposomal paclitaxel chemotherapy followed by radical hysterectomy and pelvic lymphadenectomy (NACT group) or underwent primary radical hysterectomy and pelvic lymphadenectomy (PRS group) between 2007 and 2017 were reviewed. All patients were diagnosed by cervical biopsy and pathology before treatment, and the staging was based on pelvic examination, magnetic resonance imaging (MRI), and computed tomography (CT). The patients with clinically suspected involvement of lymph nodes were subjected to primary chemoradiation. In addition, the patients who did not complete the radical surgery and those who received preoperative radiotherapy were excluded.

Clinical and pathological data including age, FIGO stage, tumor size, histology, differentiation, lymph vascular space invasion (LVSI), parametrial involvement, deep stromal invasion, surgical margin, and lymph node status were collected from the patients’ medical records.

The study was approved by the Ethics Committee of the Tongji Medical College, Huazhong University of Science and Technology (No. 2018S452), and retrospectively registered (ISRCTN24104022).

#### Management

All patients underwent radical hysterectomy and pelvic lymphadenectomy. For the NACT group, a carboplatin-liposomal paclitaxel regimen was used. Patients intravenously received paclitaxel (paclitaxel liposome for injection, Luye Pharma Group Ltd., Nanjing, China) at 135–175 mg/m^2^ on the first day and carboplatin (carboplatin for injection, Qilu Pharma Ltd., China) at an area under the curve (AUC) = 5 on the second day every 3 weeks. The number of NACT cycles ranged from 2 to 3. To achieve a better response to NACT, we initially gave the liposomal paclitaxel at a max dose and adjusted the dose according to the level of creatinine and the adverse events during the chemotherapy. Assessment of the response to NACT was based on the IMAGINE study before and after NACT according to the Response Evaluation Criteria in Solid Tumors (RECIST, version 1.1) [5]. Complete response (CR) was defined as the complete disappearance of all target lesions; partial response (PR) was defined as a ≥ 30% decrease in the sum of the diameters of target lesions; progressive disease (PD) was defined as a ≥ 20% increase in the sum of the diameters of target lesions or the appearance of new target lesions; stable disease (SD) was defined as the status between PR and PD. The NACT responders (NACT-R) were defined as patients with CR or PR, while the non-responders (NACT-NR) were defined as patients with SD or PD. After radical surgery, adjuvant therapy was recommended if patients exhibited any of the high-risk factors of lymph node metastasis, parametrial involvement, or positive surgical margins, and in patients with two or more intermediate risk factors including LVSI, deep stromal invasion (≥ 1/3 cervical stromal), and tumor size ≥ 2cm.

#### Follow-up

The follow-up was performed according to the recommendations from the National Comprehensive Center Network (NCCN) [12]. The items mainly included gynecological examination, vaginal stump cytological examination, pelvic ultrasound, and chest X-ray. The overall survival (OS) was defined as the time from completion of the operation to the death or to the date of the last contact. Progression-free survival (PFS) was defined as the time from completion of the operation to the first appearance of progressive disease or to the date of the last contact. The use of radiation and the interval between surgery and radiation (adjuvant therapy or therapy after recurrence) were used to estimate the cumulative radiation during the follow-up.

#### Statistical analysis

SPSS 23.0 (IBM Corp., Armonk, NY, USA) was used for statistical analysis. The statistical significance of variables was assessed with the *χ*^2^ test or Fisher’s exact test as appropriate. The survival curves and cumulative radiation curves were performed using the Kaplan-Meier method with GraphPad Prism 7.0 (GraphPad Software, Inc., San Diego, CA, USA), and the log-rank test was used to assess the significance of the differences. *P*-value < 0.05 was considered statistically significant.

## Results

### The characteristics of the patients

A total of 414 patients with stage IB2–IIA2 disease were included in this study. A total of 197 patients received NACT followed by radical surgery, and 217 patients received PRS. Their characteristics are listed in Table 1. The patients in the two groups were comparable in age, histological type, and tumor differentiation, but the patients in the NACT group had more advanced tumor stages and larger tumor sizes. At the end of NACT, the total response rate was 62.4%, of which 34 (17.3%) patients achieved CR and 89 (45.2%) patients achieved PR. The NACT non-responders included 71 (36.0%) patients with SD and 3 (1.5%) patients with PD.

**Table 1:** Characteristics of the patients with locally advanced cervical cancer

Characteristics	NACT, *N* (%)	PRS, *N* (%)	*P*-value (*χ*^2^ test)
Total	197	217	
Age (years)			0.051
< 45	79 (40.1)	67 (30.9)	
≥ 45	118 (59.9)	150 (69.1)	
Stage (FIGO 2009)			< 0.001
IB2	88 (44.7)	33 (15.2)	
IIA1	17 (8.6)	166 (76.5)	
IIA2	92 (46.7)	18 (8.3)	
Primary tumor size (cm)			< 0.001
< 4.0	14 (7.1)	149 (68.7)	
4.0~4.9	91 (46.2)	42 (19.4)	
5.0~5.9	59 (29.9)	23 (10.6)	
≥ 6.0	33 (16.8)	3 (1.4)	
Histology			0.356
Squamous	168 (85.3)	177 (81.6)	
Non-squamous	29 (14.7)	40 (18.4)	
Histologic grade			0.906
G1	17 (8.6)	17 (7.8)	
G2	101 (51.3)	116 (53.5)	
G3	56 (28.4)	56 (25.8)	
Unknown	23 (11.7)	28 (12.9)	

*FIGO*, International Federation of Gynecology and Obstetrics; *NACT*, neoadjuvant chemotherapy; *PRS*, primary radical surgery

### Neoadjuvant chemotherapy reduced risk factors and the need for adjuvant radiation

To investigate whether NACT could reduce the risk factors associated with recurrence and death that are prerequisites for adjuvant radiotherapy, we compared the clinicopathological risk factors between the two groups. As shown in Table 2, after NACT, the tumor volume in the NACT group significantly shrank, and there were more tumors ≤ 2.0 cm in the NACT group than in the PRS group (36.0% vs 19.8%, *P* < 0.001). The postoperative pathology examination also revealed a lower incidence of deep stromal invasion in the NACT group than in the PRS group (46.7% vs 59.5%, *P* = 0.023), whereas the two groups showed no significant differences in parametrial involvement, lymph node metastasis, LVSI, and positive surgical margin. According to the presence of pathological risk factors for recurrence, 54.82% of women in the NACT group needed adjuvant therapy, while 60.87% in the PRS group. Among them, 33.00% of NACT patients and 40.09% of PRS patients received adjuvant radiation or chemoradiation, and the others received adjuvant chemotherapy alone. In addition, 8.12% of NACT patients and 9.68% of PRS patients underwent radiotherapy after relapse.

**Table 2:** The clinicopathological risk factors in patients with locally advanced cervical cancer

Variables	NACT, *N* (%)	PRS, *N* (%)	*P*-value (*χ*^2^ test)
Total	197	217	
Preoperative tumor size			< 0.001
≤ 2.0 cm	71 (36.0)	43 (19.8)	
2.1~4.0 cm	78 (39.6)	126 (58.1)	
> 4.0 cm	48 (24.4)	48 (22.1)	
Parametrial involvement			0.776
Negative	169 (85.8)	184 (84.8)	
Positive	28 (14.2)	33 (15.2)	
Lymph node metastasis			0.336
Negative	134 (68.0)	157 (72.4)	
Positive	63 (32.0)	60 (27.6)	
LVSI			0.135
Negative	150 (76.1)	151 (69.6)	
Positive	47 (23.9)	66 (30.4)	
Cervical stromal invasion			0.023
< 1/3	40 (20.3)	36 (16.6)	
1/3~2/3	20 (10.2)	41 (18.9)	
≥ 2/3	72 (36.5)	88 (40.6)	
Unknown	65 (33.0)	52 (24.0)	
Surgery margin			0.128
Negative	190 (96.4)	202 (93.1)	
Positive	7 (3.6)	15 (6.9)	

*NACT*, neoadjuvant chemotherapy; *PRS*, primary radical surgery; *LVSI*, lymph vascular space invasion

### Survival

**Fig. 1:**
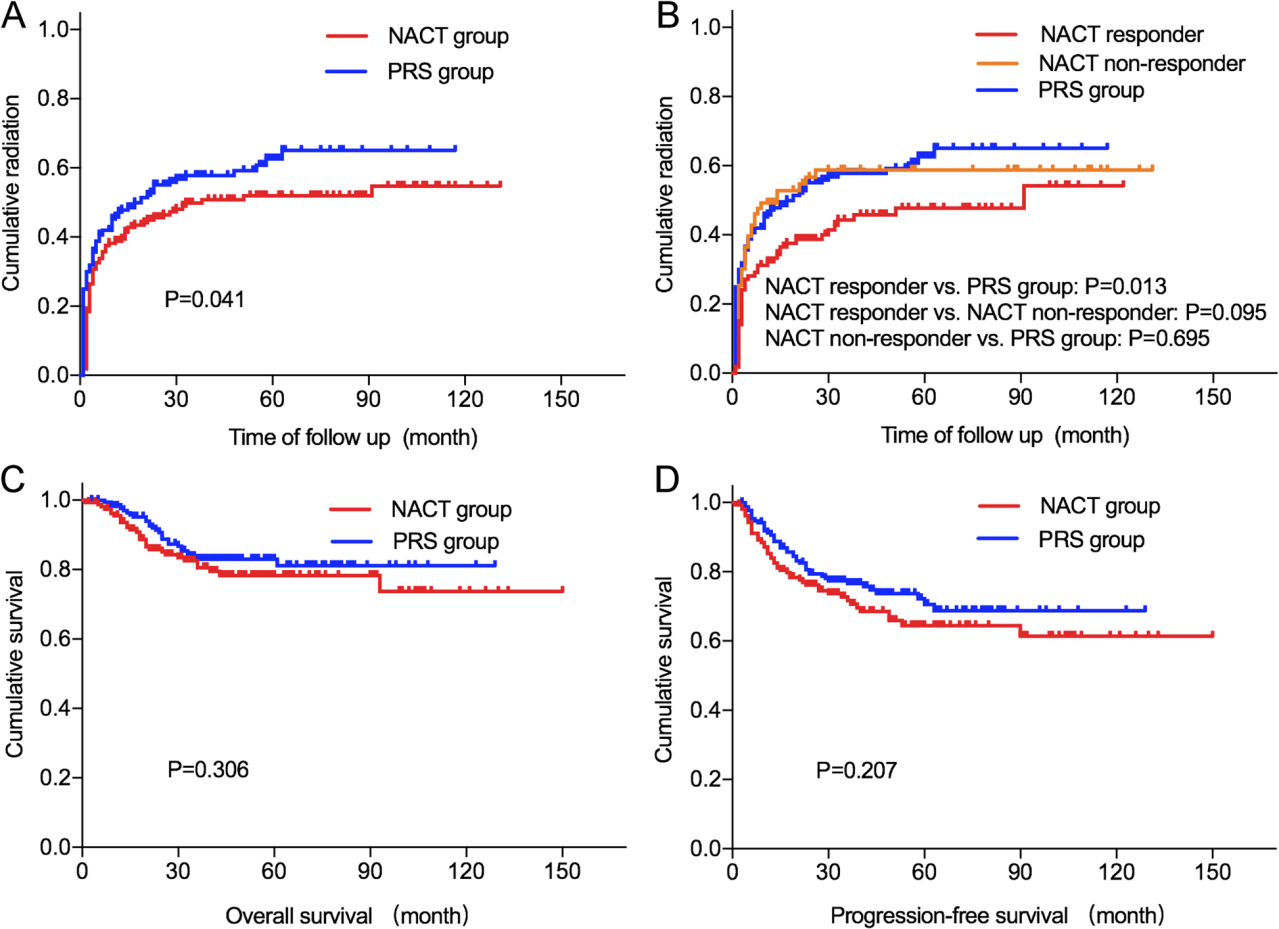
Cumulative radiation rates and survival in the NACT group versus the PRS group. The use of postoperative radiation in any setting (including adjuvant therapy after radical surgery or treatment for recurrent diseases) and the interval between surgery and radiation were used to plot the curves of cumulative radiation rates. **A** The cumulative radiation rates in the NACT group and the PRS group. **B** The cumulative radiation rates in the NACT responders, the NACT non-responders, and the PRS group. **C** The overall survival in the NACT group and the PRS group. **D** The progression-free survival in the NACT group and the PRS group. NACT, neoadjuvant chemotherapy; PRS, primary radical surgery

Furthermore, we compared the cumulative postoperative radiation rates in different groups to determine whether NACT reduced the adjuvant radiation in patients with LACC. As shown in Fig. 1A, the cumulative radiation rate of the NACT group was significantly lower than that of the PRS group (54.7% vs 65.1%; *P* = 0.041). In the subgroup analysis (Fig. 1B), the cumulative radiation rate of the NACT responders was significantly lower than that of the PRS group (54.2% vs 65.1%; *P* = 0.013), but there was no significant difference between the non-responders and PRS group (58.8% vs 65.1%; *P* = 0.695). We then compared the OS and PFS rates between the two groups to investigate whether NACT impacts the survival of patients with LACC. The median follow-up time was 45 (range, 3~150) months. The 5-year OS (78.3% vs 83.0%, *P* = 0.306) (Fig. 1C) and PFS (64.5% vs 70.6%, *P* = 0.207) (Fig. 1D) of the NACT group were comparable with those in the PRS group, suggesting that NACT did not compromise the survival.

## Discussion

As the age at which women develop cervical cancer is decreasing and radiation may have serious implications that reduce the quality of patients’ life, reducing radiation is an important challenge for patients with LACC. In this study, our results revealed that NACT with paclitaxel and carboplatin significantly reduced the clinicopathological risk factors and cumulative adjuvant radiation rate when compared to the PRS group, without compromising the survival. These findings may provide important help in the management of LACC to improve the quality of life.

Though concurrent chemoradiation is recommended as the standard treatment for patients with LACC, as the age at which women develop cervical cancer is decreasing, treatment that protects physiological function and improves the quality of life is important, and radical surgery has been chosen for LACC. However, after radical surgery, some patients still present with pelvic lymph node metastasis, parametrial involvement, positive surgery margin, LVSI, and deep stromal invasion, which are identified as risk factors for recurrence and death [7, 8], and adjuvant radiation is recommended if patients exhibit these risk factors. These patients are then faced with increased treatment and complications.

In an effort to improve the prognosis and quality of life of patients with LACC, NACT followed by radical surgery has been proposed as a promising strategy for LACC [2], which was believed to be able to reduce the risk factors of recurrence and death, and reduce the need for postoperative adjuvant radiotherapy. According to a randomized study, the pelvic lymph node metastasis (25.0% vs 42.9%, *P* = 0.025) and parametrial infiltration (25.0% vs 41.4%, *P =* 0.038) rates were significantly lower in the NACT (with cisplatin, mitomycin, and 5-fluorouracil) group than in the PRS group [1]. Yang et al. reported in their multicenter study that the incidence of LVSI and deep stromal invasion were both significantly reduced after NACT with IP and TP when compared to the PRS group (4.7% vs 18.2%, *P* = 0.002; 45.8% vs 68.2%, *P* = 0.001) [18], and Kim et al. also obtained similar results in their study with NACT consisting of paclitaxel/carboplatin, 5-fluorouracil/cisplatin, and 5-fluorouracil/carboplatin [10]. In this study, we observed that the rates of patients with tumors > 2 cm and deep stromal invasion in the NACT group were significantly lower than those in the PRS group, while there were more patients with more advanced stages and larger primary tumor sizes in the NACT group.

Regarding postoperative radiation, our results showed that the cumulative radiation rate was significantly reduced in the NACT group compared to the PRS group (54.7% vs 65.1%; *P* = 0.041), especially for the responders. Similarly, another study with NACT consisting of paclitaxel and cisplatin/carboplatin also reported that the adjuvant radiotherapy was administered to fewer patients in the NACT group compared to the PRS group [2]. Katsumata et al. revealed that the proportion of patients who met the criteria for postoperative radiation (72% vs 89%, *P* = 0.015) and patients who received postoperative radiation (58% vs 79%, *P* = 0.015) were both significantly lower in the NACT group than in the PRS group [9]. Yang et al. also reported that the rates of postoperative adjuvant radiotherapy and chemoradiation in the NACT group were lower than that of the PRS group, though without significant difference [18].

While the short-term efficacy of NACT is certain, whether NACT affects locally advanced cervical cancer patients with long-term survival remains controversial [16, 19]. Yin et al. performed a retrospective study to compare the long-term survival of NACT followed by radical surgery and primary radical surgery, and the results showed that the NACT group had significantly higher PFS (HR = 1.870, *P* = 0.0031) and OS (HR = 1.813, *P* = 0.0175) rates than the PRS group [19]. However, there were also studies that failed to obtain similar results. A phase III trial was conducted to determine whether NACT impacts the survival of LACC, and it finally failed to find any benefit for the NACT group, with similar PFS rates (56.2% vs 53.8%) and OS rates (63.3% vs 60.7%) in the NACT group compared to the PRS group [4], which was in consistence with our previous study [17]. In the present study, the 5-year OS and PFS of the NACT group (78.3% and 64.5%) were similar to those of the PRS group (83.0% and 70.6%). All these results indicated that NACT may be useful for improving the survival of patients with LACC or may confer comparable survival; it at least did not worsen the long-term survival.

Our study demonstrated the potential impact of carboplatin-liposomal paclitaxel NACT on postoperative risk factors and cumulative radiotherapy in patients with LACC. However, it had several limitations. First, despite the relatively large sample size, the single-center retrospective study design does really represent a major limitation of this study, in which selection bias and confounding bias were inevitable. Second, the patients in the NACT group and the PRS group were not very well matched at baseline, with more advanced tumors in the former group. However, despite the unbalanced tumor stage distribution in favor of PRS, the pathological and oncological outcomes of interest were comparable between the groups, suggesting that NACT is at least not inferior to PRS regarding efficacy. Third, we did not make comparisons of NACT plus radical surgery to concurrent chemoradiation, which is considered standard treatment for LACC. A recent meta-analysis including two RCTs compared NACT and surgery versus definitive concurrent chemoradiation in terms of survival and acute toxicity in patients with LACC (stages IB2, IIA, and IIB) [6]. The results showed that patients in the concurrent chemoradiation group had superior DFS and less acute toxicity, but with low-grade evidence. The firmest conclusion drawn from this meta-analysis is that NACT + surgery and CRT have similar OS benefits, suggesting NACT + surgery is a reasonable alternative to the standard, and its benefits are worth further detailed investigation in terms of long-term adverse effects and quality of life.

In conclusion, our results suggest that carboplatin-liposomal paclitaxel NACT before RS can reduce the rate of patients who received postoperative radiotherapy, without compromising the survival.

## Data Availability

Data are available within the article or its supplementary materials. Other data that support the findings of this study are available from the corresponding authors on reasonable request.
